# Leading by example: Experimental evidence that therapist lived experience disclosures can model the path to recovery for clients

**DOI:** 10.1111/bjop.12759

**Published:** 2025-01-11

**Authors:** Alysia M. Robertson, Tegan Cruwys, Mark Stevens, Michael J. Platow

**Affiliations:** ^1^ School of Medicine and Psychology The Australian National University Canberra Australian Capital Territory Australia

**Keywords:** depression, group psychotherapy, lived experience, social identity, therapist self‐disclosure

## Abstract

A common guideline for self‐disclosure is that therapists should only share *recovered* personal experiences with clients (i.e., no longer distressing). However, theoretical rationale and empirical support for this claim is limited. Drawing on identity leadership theorizing, we investigated whether recovery disclosures are beneficial to the extent that they signal a therapist's *aspirational prototypicality* (i.e., embodiment of an aspirational identity for clients). Across two experimental studies (*N* = 545), we recruited clients, therapists and general population adults. Participants read a group therapy for depression vignette in which the therapist disclosed: nothing, professional experience with depression, current depression, recovered depression or recovered anxiety. Participants rated the prototypicality of the therapist, the extent to which they perceived the therapist positively, the therapist's expertness and the expected prognosis for therapy. Contrary to our hypotheses, the type of disclosure did not significantly affect positive perceptions, expertness or expected prognosis ratings. However, the therapist disclosing a recovered and relevant condition (recovered depression) was rated as significantly more aspirationally prototypical than the other therapists. Given prior evidence that group therapists are more effective when viewed as aspirationally prototypical, our findings suggest that recovery disclosures may represent one way therapists can signal their prototypicality and enhance their effectiveness.

## BACKGROUND

Most therapists would agree that destigmatizing mental health issues is an important endeavour. One way that therapists could help reduce stigma is by honestly discussing their own lived experience of mental ill health (something that many therapists have; Victor et al., 2022). Ironically, however, many therapists choose not to disclose this information to clients or other therapists precisely because of fears about stigma (Harris et al., 2019; McPhee et al., 2023; White et al., 2006). Indeed, this fear may be well justified, with evidence suggesting that even researchers who conduct self‐relevant research on mental health (so‐called ‘me‐search’) are often perceived negatively (Devendorf et al., 2023).

Several other factors may also prevent therapists from disclosing. For example, in some countries (e.g., Australia), regulatory bodies mandate that health professionals report other professionals who are ‘impaired’ (Australian Health Practitioner Regulation Agency, 2023). Therapists may therefore consider disclosing mental health conditions a risk to their livelihood. Another possible reason not to disclose relates to concerns about client welfare: therapist self‐disclosure has been described as a ‘slippery slope’ that can lead to boundary violations, harmful multiple relationships and role confusion (Barnett, 2011; Strasburger et al., 1992).

Despite these stark warnings, however, evidence for the ‘dangers’ of self‐disclosure is limited and provided primarily by extreme case scenarios rather than targeted empirical investigations. A more robust body of research speaks to the potential *benefits* of therapist self‐disclosure and, more broadly, mental health care delivered by people with lived experience (Conchar & Repper, 2014; Henretty et al., 2014; Henretty & Levitt, 2010). Therapists (as well as researchers and teachers in psychology) with lived experience therefore need to carefully weigh up the potential benefits and costs of disclosing this experience to clients, students or colleagues (see Hinshaw, 2008).

We aimed to formally evaluate how therapists who disclose their own experience with common mental health conditions are perceived by clients, other therapists and adults in the general population. More specifically, we examined experimentally whether the potential benefits of such disclosures vary as a function of whether therapists disclose that they have *recovered from* or are *still experiencing* the condition. To further advance understanding, we also sought to shed light on the psychological mechanisms through which the benefits of therapist disclosure operate.

### What should therapists disclose about their mental health and when?

Part of the reason for the lack of consensus on therapist self‐disclosure may be that its effects depend on what and how much is disclosed. To assist therapists, some researchers have proposed guidelines for self‐disclosure. One common recommendation is that therapists should not disclose anything that is *currently* distressing to them (Gelso & Palma, 2011; Henretty & Levitt, 2010; Knox & Hill, 2003; Levitt et al., 2016). This recommendation has recently received some empirical support. Moody et al. (2021) asked 417 adults to read one of four vignettes in which therapists disclosed either: (a) nothing, (b) a mental health condition (bipolar disorder or depression) that happened in the distant past, (c) a mental health condition from the recent past or (d) a mental health condition that they were currently experiencing. The researchers found that therapists who recounted a mental health condition from the distant past were rated more favourably than both the non‐disclosing therapist and the therapist currently experiencing a mental health condition.

Similarly, in another between‐participants vignette study, Somers et al. (2014) asked undergraduates to evaluate a therapist who either did or did not disclose their prior experience with a psychological issue (depression, post‐traumatic stress disorder or alcohol dependence). Across all three presenting problems, participants rated the disclosing therapist more positively than the non‐disclosing therapist. Participants also perceived the disclosing therapist to have a better working relationship with the client and were more confident that treatment would be successful. In another vignette study, McCormic et al. (2019) found that undergraduates rated therapists most favourably when they disclosed a moderate amount about a previous mental health condition (i.e., listed their symptoms but did not talk about negative impacts on their life). This provided some support for Gelso and Palma's (2011) earlier claim that therapist self‐disclosure is beneficial for clients only up to a certain level of frequency and intensity (i.e., an inverted U relationship).

These studies provide evidence that therapist disclosure can be received positively. However, they have some notable limitations. The samples consisted of adults from the general population or students, raising questions about the external validity of the findings. It is possible, for example, that recent therapy clients may perceive disclosures more positively than the general community because they may view disclosing therapists as more similar to themselves in important ways. The opinions of therapists themselves regarding the appropriateness of therapist disclosure may also differ from these general community samples. Many therapists choose to keep their mental health issues secret from clients and colleagues due to fears that they will be judged or appear unprofessional (e.g., Victor et al., 2022; White et al., 2006), and thus may rate therapists who choose to disclose more negatively. Most importantly, research in this area has lacked a theoretical framework to guide investigations into why and how therapist self‐disclosure might affect outcomes.

### A new theoretical framework for self‐disclosure: identity leadership theory

One framework that may help shed light on therapist self‐disclosure is identity leadership theory (Haslam et al., 2020). This theory builds on the social identity approach, which proposes that people can, and do, define themselves not only as unique individuals (as ‘I’ and ‘me’) but also in terms of the social groups to which they belong (e.g., as ‘us’ members of this therapy group; Tajfel & Turner, 1979). According to the social identity approach, when people define themselves in terms of a particular group membership that they share with others, this lays the foundation for processes of social influence (Turner, 1991). The word ‘leadership’ is rarely applied in therapy contexts. However, like those responsible for leading groups in other contexts (e.g., business or sport), therapists seek to influence people (i.e., their clients) to change in positive ways and take steps toward important goals (Platow et al., 2020; Robertson et al., 2023). Identity leadership theory provides novel insights into how this influence operates through shared social identities, positing that a leader's ability to achieve influence is tied to how successful they are in building a shared connection with those they lead.

The theory proposes four key processes through which leaders can build a sense of shared social identification among their followers and, consequently, achieve influence (see Haslam et al., 2020). First, *identity prototypicality* (‘prototypicality’ henceforth) refers to how well leaders embody important aspects of the group's identity (e.g., by disclosing ways that they are similar to the group). Second, *identity advancement* refers to how well leaders promote the group's collective interests and act as a ‘champion’ for the group (e.g., by advocating for the unique needs of the group). Third, *identity entrepreneurship* refers to how well leaders actively craft and strengthen the specific identity of the group (e.g., through setting explicit norms and rules for the group). Finally, *identity impresarioship* refers to how well leaders create structures and opportunities for group members to express and develop their identity (e.g., through organizing relevant activities).

**FIGURE 1:**
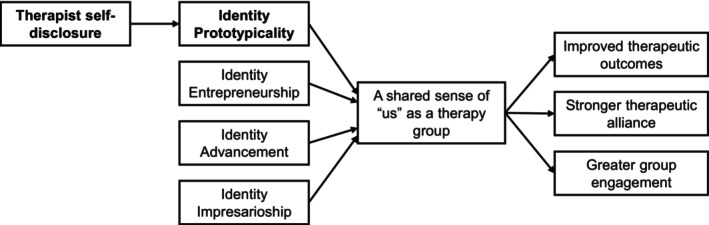
Conceptual model of identity leadership in group therapy. Example indicative references: Cruwys et al. (2023), Lee et al. (2023), Robertson et al. (2022), Stevens et al. (2022). We were primarily focused on testing the bolded path in this study.

Through these four processes, identity leadership theory provides a framework for understanding multiple therapy‐related outcomes. Some examples of these are presented in Figure 1. The primary hypothesised mechanism through which these positive outcomes occur is enhanced social identification with the therapist or therapy group. However, other social psychological processes also play key roles (e.g., the development of health‐promoting norms in the therapy group; Robertson et al., 2022). Although we provide some examples of benefits that identity leadership might confer via social identification with an emergent therapy group identity, a much larger body of research speaks to the broader mental and physical health benefits of experiencing social identification with meaningful groups (Haslam et al., 2018; Jetten et al., 2017). For example, social identification has been linked to greater resilience (Koni et al., 2019), reduced depression (Postmes et al., 2019), improved post‐traumatic symptoms (Muldoon et al., 2019) and more general psychological resources, such as a sense of belonging, control, hope and purpose (Frings et al., 2019; Greenaway et al., 2016; Walter et al., 2016). According to this hypothesized model (see also Lee et al., 2021; Robertson et al., 2023), therapist characteristics and behaviours—including carefully considered lived experience disclosures—can influence relevant outcomes by impacting important identity‐based group processes.

It is worth noting that these mechanisms align closely with established therapeutic processes. In particular, common factors theory posits that different therapeutic approaches all share several ‘common factors’ that determine their effectiveness, including positive therapeutic relationships between therapists and clients (Messer & Wampold, 2002; Wampold, 2001, 2015). The therapeutic relationship is commonly termed the therapeutic alliance in individual therapy (i.e., the bond between the therapist and client; Horvath et al., 2011) and group cohesion in group therapy (i.e., the bond between the therapist and the group members but also *among* the group members; Burlingame et al., 2018). Alliance and cohesion have long been recognized as key to unlocking positive therapeutic outcomes (Ardito & Rabellino, 2011; Burlingame et al., 2018).

Compared to these existing understandings of the therapeutic relationship, identity leadership theory: (a) offers new perspectives on how the therapeutic relationship is formed and operates, drawn from a long tradition of robust theory and research (i.e., since Tajfel & Turner, 1979) and (b) provides insights on specific therapist behaviours that might strengthen the therapeutic relationship (e.g., see tables in Lee et al., 2021; Robertson et al., 2023). According to identity leadership theory, the key to effective alliance or cohesion is that therapists and clients connect over meaningful, shared parts of their identity (Lee et al., 2021; Robertson et al., 2023). This proposition has also received empirical support: clients' social identification with the therapist and ratings of their therapist's identity leadership were shown in recent studies to predict substantial variance in the therapeutic alliance (Cruwys et al., 2023; Lee et al., 2023). Critically, according to the theory, it is not enough for a therapist to be simply appointed as a leader or to be an exceptional individual. Instead, influence depends on an emergent sense of a collective psychological community that includes both client(s) and therapist(s). Given this theoretical focus on the collective, we primarily focus on group therapy contexts in this article. However, conceptually, this approach is equally applicable to individual therapy, such that the therapist and client can bond over broader shared identities.

Importantly, the basis of this collective sense of ‘us’ as therapist and client(s) extends beyond superficial similarities that prior research has focused on, such as therapists and clients being from the same ethnic or racial group. Indeed, although it has intuitive appeal, meta‐analytic evidence has indicated that simple demographic matching between therapists and clients has minimal therapeutic benefit (Cabral & Smith, 2011). Rather than assuming that similarity between the therapist and clients will necessarily lead to a strong therapeutic relationship, identity leadership theory emphasizes the active role of the therapist in developing a meaningful shared therapy identity. This identity encompasses collective values, norms and behaviours that facilitate therapeutic change and emerge through active processes of identity development and leadership by the therapist (see Lee et al., 2021 for further discussion). Through this lens, effective therapy relationships are built not on demographic matching or incidental similarities, but on cultivating a *purposeful* therapeutic identity, oriented toward recovery and mental health. A key benefit here is that directing therapists to cultivate new, therapy‐based social identities is a far more actionable (and feasible) approach than ensuring that therapists are matched to clients on key demographic factors, which may be especially difficult in group settings.

Although it has not yet been tested, the prototypicality dimension of identity leadership appears particularly relevant to understanding the benefits of lived experience disclosures. There are at least two different ways that therapists could present themselves as prototypical to clients. On one hand, a therapist could seek to show that they represent ‘who we are now’ (*average prototypicality*) – for example, by disclosing that they are currently experiencing the same mental health condition as group members. On the other, a therapist could seek to represent ‘who we want to be’ (*aspirational prototypicality*) – for example, by disclosing lived experience of *recovering from* the same mental health condition as group members. A recent meta‐analysis of studies conducted primarily in organizational contexts found that, while there was evidence for the benefits of both types of prototypicality, aspirational prototypicality was a stronger predictor of positive outcomes than average prototypicality (Steffens et al., 2021). In a group therapy context, Robertson et al. (2022) similarly found that clients' ratings of their group facilitators' aspirational prototypicality positively predicted desirable normative change across therapy. Specifically, in the context of an eating disorder prevention group, the extent that clients perceived their group facilitators to have a positive body image (compared to their fellow group members) led to increased reductions in the group's approval of unhealthy dieting practices across therapy. Prototypicality also indirectly predicted positive therapeutic outcomes via this normative change, including reduced intentions to diet in the three months post‐therapy and greater body satisfaction.

### The present research

Building on the abovementioned evidence for the benefits of group therapy leaders being prototypical, we examined whether therapist lived experience disclosures could be used to signal a therapist's prototypicality. We conducted two experiments to examine how therapists' disclosure of recovered or current mental health conditions affected perceptions of their prototypicality as well as more general perceptions of them and their effectiveness. Our samples included therapy clients (Studies 1 and 2), therapists (Study 1) and general population adults (Study 2). Participants were randomly assigned to read a group therapy for depression vignette in which we varied what the therapist disclosed. Across both studies, we examined three key outcome variables: (1) positive perceptions of the therapist, (2) perceived expertness of the therapist and (3) expected prognosis for therapy (which has been associated with better treatment outcomes; Constantino et al., 2018). We chose depression as the disclosure context to maximize comparability between our results and those of previous studies on this topic (McCormic et al., 2019; Moody et al., 2021) and because depression is one of the most common mental health conditions with the highest associated burden worldwide (Institute of Health Metrics and Evaluation, 2023). Despite this focus on depression, we did not specify experience with depression as part of our inclusion criteria. This decision was made so that we could examine a wider range of perspectives on therapist self‐disclosure of lived experience, rather than only sampling therapists, clients and general population adults with a history of depression. However, to gain some insight into participants' level of connection to the vignettes, in Study 1 we examined participants' connection with depression. Specifically, we measured their current depression symptoms, previous experience of depression and identification as depressed as covariates of interest.

## STUDY 1

In Study 1 we recruited a sample of clients and therapists to examine whether participants' perceptions of therapists differed across four scenarios: (1) the therapist discloses nothing about personal experiences with depression, (2) the therapist discloses years of *professional* experience with depression, (3) the therapist discloses years of *personal*, *previous* experience with depression or (4) the therapist discloses years of *personal*, *current* experience with depression. We predicted that a therapist disclosing previous experience with depression to a therapy group for people with depression would be perceived as more representative of the group's aspired identity (‘who we want to be’), while a therapist disclosing current experience of depression would be perceived as more representative of the group's current identity (‘who we are’). Our hypotheses (preregistered prior to data collection; https://osf.io/az2v9/) were as follows:

The therapist disclosing *recovered* depression would be rated most favourably on positive perceptions (H1a), expertness (H1b) and expected prognosis for therapy (H1c). We also expected that the non‐disclosing therapist would be rated least favourably.

As this was the first empirical study to examine therapist self‐disclosure of a mental health condition in an intentional sample of real clients and real therapists, we were also interested in whether ratings of the therapist differed between the client and therapist samples. Given warnings against therapist self‐disclosure and the stigma that surrounds health professionals with lived experience, we hypothesized the following:

Clients would rate therapists who disclose information about their mental health condition (current or recovered depression) significantly more favourably than therapists on positive perceptions (H2a), expertness (H2b) and expected prognosis for therapy (H2c).

Finally, in line with identity leadership theorizing, we hypothesized the following:

The therapist disclosing recovered depression would be rated significantly higher on aspirational prototypicality (representing ‘who we want to be’) than therapists in the other conditions (H3a). Along these lines, we also hypothesized that the therapist disclosing current depression would be rated significantly higher on average prototypicality (representing ‘who we are’) than therapists in the other conditions (H3b).

Given that the context of the vignettes was disclosure of depression, we also pre‐registered our plans to conduct several exploratory analyses examining the effect of participants' depression (past and current symptoms) and identification as a depressed person as covariates but did not make any specific hypotheses about these effects.

### Study 1 Material and methods

#### Participants

We recruited two subsamples: 160 clients who had received therapy within the last year and 160 therapists who had delivered mental health care services within the last year. The samples were primarily recruited via the research platform Prolific and were identified using a paid screener questionnaire. The screener included distractor items in addition to those designed to establish eligibility. This made it such that our specific eligibility criteria were unclear to participants and they were not incentivized to lie to gain access to the full study (Chandler & Paolacci, 2017). Participants were paid the standard Prolific rate (i.e., £2.70 or equivalent AUD for their time completing the full survey plus £0.17 for completing the screener). For the client subsample, people whose Prolific profile indicated that they had attended a healthcare provider consultation and/or received NHS mental health support in the past year were eligible to complete the screener. Of the 270 people who completed the screener, 195 indicated that they had received mental health services in the last year (i.e., answered yes to the question: ‘Have you received mental health care services in the last 12 months?’) and were invited to complete the full study (160 did). For the therapist subsample, people whose Prolific profile indicated that they worked in the ‘health care and social assistance’ or ‘medical/healthcare’ industry were eligible to complete the screener. Of the 470 people who completed the screener, 175 indicated that they had delivered mental health services in the last year (i.e., answered yes to the question ‘Have you provided mental health care services to a client in the last 12 months?’) and were invited to complete the full study (150 people did). An additional 10 therapists were recruited via snowball sampling (i.e., known health professionals were invited via personal networks).

We aimed to recruit therapists and clients to better generalize our findings to the target population: people currently engaged in delivering or accessing therapy, who may therefore be the providers or recipients of a therapist lived experience disclosure. To provide a larger recruitment pool, we defined therapy broadly in our eligibility criteria, encompassing any mental health service in which clients receive mental health support from a health professional. In the screener, we provided participants with examples of what these services might entail (therapy, counselling, discussing mental health symptoms). Given this broad definition, participants likely delivered and accessed a variety of services, ranging from structured psychotherapy (e.g., in the case of a psychologist) to more informal or brief forms of support (e.g., a mental health consultation with a general practitioner).

The target sample size was determined using an a priori power analysis in G*Power (Faul et al., 2007) and preregistered. We calculated the sample size to detect an effect size of *η* ^2^ = .08 (drawn from McCormic et al., 2019) with a power of .85 in a one‐way fixed effects ANOVA with four groups. This suggested that the minimum sample size should be 152 (which we rounded up to 160). We aimed to recruit 160 therapists and 160 clients to provide adequate power for comparisons within each sub‐sample.

All participants completed an attention check asking them about the primary symptom experienced by the group in the vignette they read (i.e., depression) and a manipulation check asking them to identify what was disclosed by the therapist. Participants in the therapist subsample were also asked to describe their role to ensure that they were working as a therapist. These checks led to the removal of 38 participants (19 clients, 19 therapists): 32 failed the manipulation check, four failed the attention check and two from the therapist subsample wrote that they were not therapists. We did not exclude two people who chose not to answer the question about their role. The final sample after these exclusions was 282 (141 clients and 141 therapists).

The two subsamples had comparable demographic characteristics (see Table 1). All participants resided in the United Kingdom except for the 10 therapists recruited via snowball sampling, who resided in Australia. The therapist participants were drawn from a range of professions: psychotherapist/counsellor (*n* = 29), social worker (*n* = 27), mental health nurse/nurse (*n* = 24), mental health support worker/support worker (*n* = 16), psychologist (*n* = 15) and other healthcare worker (e.g., midwife, intake worker, speech therapist, occupational therapist; *n* = 20). An additional eight therapists specified multiple roles (e.g., counsellor and nurse) and two did not specify their role. Years of experience ranged from <1 to 40 years (*M* = 10.84 years, *SD* = 8.40).

**TABLE 1:** Study 1 sample demographics.

Subsample	Mean age (*SD*)	Gender, % (*n*)	Ethnicity, % (*n*)
Therapists (*N* = 141)	41.50 (11.99)	Women = 70.2% (99) Men = 28.4% (40) Non‐binary <1% (1) Rather not say <1% (1)	White = 85.1% (120) Black = 5.0% (7) Asian = 6.4% (9) Mixed = 3.5% (5)
Clients (*N* = 141)	38.43 (10.47)	Women = 71.6% (101) Men = 27.7% (39) Non‐binary <1% (1)	White = 90.8% (128) Black = 2.1% (3) Asian = 2.1% (3) Mixed = 3.5% (5) Middle Eastern <1% (1) Latin(x) <1% (1)

#### Procedure and materials

Participants were randomly assigned to read one of four scenarios (adapted from McCormic et al., 2019 and very similar to those used in Moody et al., 2021). Each scenario began with,

Imagine you are in your first session of group therapy for the treatment of depression. Your group members are discussing what it feels like to have depression: a low mood, loss of interest in activities once enjoyed, trouble sleeping, feelings of worthlessness, and decreased appetite. The therapist explains the process of group therapy and how the group will work together to help decrease these depressive symptoms.

These sentences were almost identical to McCormic et al.'s (2019), with the exception that the scenario referred to group, rather than individual, therapy. We also did not add the final sentence of their opening section (i.e., ‘They tell you that they have many years of experience working with other clients like yourself and they are confident in their ability to help you’) as we deemed this a type of disclosure (professional experience). Different sentences were added depending on the experimental condition (see Table 2). These sentences were constructed to be directly comparable across conditions. All referred to ‘years of experience’, with the key difference being whether the therapist was disclosing years of professional experience, current depression or recovered depression. After reading their vignette, participants completed the measures below and provided demographic information. Informed consent was obtained from all participants in both studies. The ethical aspects of both studies were approved by the authors' university Human Research Ethics Committee (#2022/795).

**TABLE 2:** Details of scenarios provided to participants.

Scenario	Sentences added
Control	None
Therapist discloses professional experience	*The therapist also tells the group that they have many years of experience working with people with depression. The therapist says that this means they really understand how difficult it is to live with depression*
Therapist discloses recovered (previous) lived experience of depression	*The therapist also tells the group that they have personal experience with depression but are now recovered after many years of struggling. The therapist says that this means they really understand how difficult it is to live with depression*
Therapist discloses current lived experience of depression	*The therapist also tells the group that they have personal experience with depression and are currently experiencing it. The therapist says that this means they really understand how difficult it is to live with depression*

#### Measures

##### Positive perceptions

Participants' perceptions of the therapist were measured using Somers et al.'s (2014) scale (see also McCormic et al., 2019; Moody et al., 2021). Although the scale has not been formally validated, it has high internal reliability (Study 1 *α* = .93; Study 2 *α* = .92). The scale consists of nine items (e.g., ‘I think the psychotherapist is likeable’) rated on a five‐point scale (1 *Strongly Disagree* – 5 *Strongly Agree*). We added one item to examine the extent to which participants viewed the therapist as professional (i.e., ‘I think the psychotherapist is professional’).

##### Expertness

The extent to which participants viewed the therapist as an expert was measured using the expertness subscale of the Counsellor Rating Form—Short Version (CRF‐SF; Corrigan & Schmidt, 1983). Participants rated the therapist on four adjectives (Expert, Experienced, Prepared, Skilful) on four‐point scales (1 *Not Very* – 7 *Very*). This scale has been validated in prior research (Tracey et al., 1988) and had excellent internal reliability in the present study (Study 1 *α* = .94; Study 2 *α* = .92).

##### Prognosis

Expected treatment prognosis was measured using the Expectations for Treatment Scale (Barth et al., 2019). This consists of five items (e.g., ‘I expect the treatment (group therapy) would help me to cope with my complaints’) rated on a four‐point scale (1 *Partially disagree –* 4 *Definitely agree*). This measure has been validated and has excellent internal reliability (Barth et al., 2019; Study 1 *α* = .90; Study 2 *α* = .88). We found the wording of one tem (i.e., ‘I expect the treatment would improve my physical problems’) less relevant for this study, so changed it to refer to ‘mental health’. We also adapted the phrasing for the therapist sample to refer to a client rather than themselves.

##### Prototypicality of the therapist

Prototypicality was measured using a modified version of the prototypicality subscale of the Identity Leadership Inventory (van Knippenberg et al., 2024). This version includes two subscales of six items each. The first measures average prototypicality – how much a leader represents a group's *current* identity (e.g., ‘The therapist is very similar to the members of the therapy group’). The second measures aspirational (also called ‘ideal’) prototypicality—how much a leader represents a group's aspired identity (e.g., ‘This therapist embodies what the therapy group stands for’). Items are rated on a seven‐point scale (1 *Not at all* — 7 *Completely*). Both the average prototypicality subscale (Study 1 *α* = .95; Study 2 *α* = .93) and the aspirational subscale demonstrated excellent internal reliability (Study 1 *α* = .94; Study 2 *α* = .93).

##### Depression

To measure participants' current levels of depression, we used the depression subscale of the Depression Anxiety and Stress Scales‐21 (DASS; Lovibond & Lovibond, 1995). This consists of seven items (e.g., ‘I couldn't seem to experience any positive feeling at all’), which are rated on a four‐point scale (0 *Did not apply to me at all* – 3 *Applied to me very much or most of the time*). Scores are summed, then multiplied by 2 (due to the scale being a shortened version of the DASS‐42), such that higher scores indicate greater levels of depression. The scale had excellent internal reliability in the present study (Study 1 *α* = .94). We also asked, ‘Have you ever been diagnosed with depression by a health professional?’ (Yes, No, Unsure, Rather not say).

##### Social identification as depressed

We measured participants' social identification with the identity of being a ‘depressed person’ using the scale in Cruwys and Gunaseelan (2016). The scale consists of 11 items (‘I feel a bond with other people who have depression’) rated on a seven‐point scale (1 *Strongly Disagree* – 7 *Strongly Agree*). The scale had good internal reliability (Study 1 *α* = .89).

### Study 1 Results

Only six of the 282 participants had any missing data (2.1%). In most cases, these participants were only missing data for one or two items in a scale, so we calculated each measure based on the items they did complete rather than using listwise deletion. Unless otherwise specified, this means that all participants (*N* = 282) were included in all analyses. The assumptions for an ANOVA (the primary analysis to test our hypotheses) were met with minor exceptions for all variables except for expertness, which demonstrated significant violations of normality in all four conditions due to negative skew and leptokurtosis (Shapiro–Wilk tests, *p*s < .05). A Levene's test also indicated violations to homogeneity of variance, *F*(3, 278) = 6.09, *p* < .001. Where available, we therefore used non‐parametric tests where expertness was the outcome. Means, standard deviations and correlations for each measure are summarized in Table 3. Means and standard deviations of each variable by condition are shown in Table 4. We also used ANOVA and Chi‐square tests to examine whether there were any baseline differences between participants in the experimental conditions on age, gender and ethnicity. As these were non‐significant, we did not control for these variables in our analyses.

**TABLE 3:** Descriptive statistics of Study 1 key variables.

Variable	Mean	*SD*	1	2	3	4	5	6
1. Average prototypicality	4.12	1.40	1					
2. Aspirational prototypicality	4.57	1.38	.59***	1				
3. Positive perceptions	3.87	0.72	.41***	.68***	1			
4. Prognosis for treatment	2.34	0.68	.31***	.50***	.62***	1		
5. Expertness	5.20	1.20	.26***	.65***	.81***	.56***	1	
6. Depression (DASS)	14.50	11.75	.03	.05	.09	−.14*	.12	1
7. Identification as depressed	4.02	1.02	.24***	.35***	.44***	.39***	.39***	.16**

*Note*: According to Funder and Ozer (2019), the effect size of Pearson's *r* values can be interpreted such that *r* = .05 is very small, *r* = .10 is small, *r* = .20 is medium, *r* = .30 is large.

Abbreviation: *SD*, standard deviation.

* *p* < .05.

** *p* < .01.

*** *p* < .001.

**TABLE 4:** Means and standard deviations (*SD*s) of Study 1 outcome variables by condition.

Variable	Control		Professional experience		Current depression		Recovered depression	
	Mean	*SD*	Mean	*SD*	Mean	*SD*	Mean	*SD*
Positive perceptions	3.90	0.62	3.85	0.69	3.75	0.82	3.97	0.74
Expertness	5.35	1.05	5.31	0.99	4.80	1.55	5.31	1.10
Prognosis	2.32	0.66	2.31	0.69	2.24	0.71	2.46	0.65
Aspirational prototypicality	4.44^a^	1.13	4.02^a^	1.33	4.40^a^	1.38	5.37^b^	1.26
Average prototypicality	3.42^c^	1.07	3.19^c^	1.11	5.03^d^	1.27	4.82^d^	1.09

*Note*: Means with different superscript letters differ significantly, all *p* < .001.

#### Covariates

Using ANCOVA and moderation analysis (i.e., testing the effect of condition on key outcome variables with depression added as a covariate) and bootstrapped regression for the expertness variable, we found that participants' depression was not a significant covariate or moderator in any model, nor did it impact any of the key results. We, therefore, do not report the results controlling for current or past depression. We report the results of models predicting the outcome variables with social identification as depressed added as a moderator below. We compared the level of depression in the therapist and client samples. Clients reported greater current depression (*M* = 20.26, *SD* = 11.51) than therapists (*M* = 8.75, *SD* = 8.84), *t*(280) = −9.41, *p* < .001. According to Lovibond and Lovibond (1995), the mean depression score of ~20 indicates a moderately severe level of clinical depression among clients, while the mean score of ~9 we observed for therapists is in the normal – mild depression range. Additionally, 86.5% of clients reported experiencing depression in the past, compared to 51.8% of therapists.

#### H1: Difference between disclosure types

To test our hypothesis that participants would rate the therapist disclosing they had recovered from depression more favourably than the other therapists, we conducted two one‐way ANOVAs, with positive perceptions and expected prognosis as the outcome variables. For expertness, we ran a Kruskal–Wallis test. Results are summarized in Table 5 (see also Table 4 for means and standard deviations of each condition). There were no significant differences between the four conditions for any of the outcome variables and thus H1a–H1c were not supported.

**TABLE 5:** Omnibus effect of condition (disclosure type) on positive perceptions, expertness, and prognosis in Study 1.

Model term	*df*	Positive perceptions (H1a)			Expertness (H1b)		Prognosis (H1c)		
		*F*	*p*	*η* ^2^	*H*	*p*	*F*	*p*	*η* ^2^
Condition	3, 278	1.166	.323	.01	4.987	.173	1.249	.292	.01

*Note*: Tests of H1a and H1c are ANOVAs, H1b is a Kruskal–Wallis test. According to Cohen (1988), the effect size for these *η* ^2^ values is small (i.e., *η* ^2^ = .01).

#### H2: Client versus therapist ratings

To test whether the client and therapist samples differed in their ratings of the therapists, we specified an interaction term between sample (client vs. therapy) and condition (a) in two‐way ANOVAs (for the positive perceptions and prognosis dependent variables) and (b) in a bootstrapped regression specifying 1000 repetitions (for the expertness dependent variable; Fox, 2008; Pek et al., 2018). Results are summarized in Table 6. The hypotheses were not supported: no significant interactions were observed between condition and sample for positive perceptions of the therapist (H2a), expertness (H2b) or expected prognosis (H2c). However, there was a significant main effect for expected prognosis, such that prognosis was rated more positively by therapists (*M* = 2.51, *SD* = 0.62) than clients (*M* = 2.15, *SD* = 0.69).

**TABLE 6:** Effect of sample, condition and their interaction on Study 1 key outcome variables.

Model term	*df*	Positive perceptions (H2a)			Expertness (H2b)		Prognosis (H2c)		
		*F*	*p*	*η* ^2^	*χ* ^2^	*p*	*F*	*p*	*η* ^2^
Condition (disclosure type)	3, 274	1.068	.363	.01	2.24	.524	1.285	.280	.01
Sample (therapist or client)	1, 274	0.877	.350	.003	0.15	.700	20.726	<.001	.07
Condition × Sample	3, 274	1.318	.269	.01	4.25	.236	0.270	.847	.003

*Note*: *η* ^2^ reported are partial (i.e., the variance explained by each predictor partialling out the others). *χ* ^2^ values represent omnibus test of the joint significance of all levels of the variable. According to Richardson (2011), the effect size for partial *η* ^2^ can be interpreted in line with Cohen's cutoffs for *η* ^2^ (small = .01, medium = .06, large = .14).

#### H3: Type of prototypicality

To test the effect of therapist disclosure type on aspirational prototypicality (H3a) and average prototypicality (H3b), we used one‐way ANOVAs with prototypicality (aspirational or average) as the outcome and condition as the predictor. Results are summarized in Table 7 (see Table 4 for means in each condition). H3a was supported: Mean comparisons revealed that the therapist disclosing recovered depression was rated as significantly more aspirationally prototypical compared to (a) the therapist disclosing current depression, (b) the therapist disclosing years of professional experience and (c) the control therapist. H3b was partially supported: Mean comparisons revealed that the therapist disclosing current depression was rated significantly higher on average prototypicality than both the therapist disclosing years of professional experience and the control therapist, but not compared to the therapist disclosing recovered depression.

**TABLE 7:** Effect of condition (disclosure type) on prototypicality in Study 1.

Prototypicality type	*df*	Condition		
		*F*	*p*	*η* ^2^
Aspirational (H3a)	3, 277	15.097	<.001***	.14
Average (H3b)	3, 278	49.031	<.001***	.35

*Note*: ***p < .001; *N* = 280 for H3a model due to one participant missing data on all items. According to Cohen (1988), the effect size for *η* ^2^ can be interpreted such that *η* ^2^ = .01 is a small effect, *η* ^2^ = .06 is a medium effect, and *η* ^2^ = .14 is a large effect.

#### Exploratory analyses

##### Relationship between prototypicality and outcomes

We did not make any a priori hypotheses about the relationships between the prototypicality measures and key outcomes (positive perceptions of the therapist, expertness and prognosis). However, given the theoretical relevance of these relationships, we tested them in three regressions, one for each dependent variable and with average and aspirational prototypicality as independent variables (collinearity between prototypicality types was acceptable, VIF = 1.53, tolerance = 0.66; Hair et al., 2010). For the expertness variable, the regression was bootstrapped specifying 1000 repetitions to handle the violations of normality. The overall regressions were significant for positive perceptions, *F*(2, 278) = 115.73, *p* < .001, *R* ^2^ = .45; expertness, *F*(2, 278) = 106.19, *p* < .001, *R* ^2^ = .43 and prognosis, *F*(2, 278) = 45.74, *p* < .001, *R* ^2^ = .25. According to Cohen (1988), the *R* ^2^ values for positive perceptions and expertness indicate a large effect size (>.26), and the *R* ^2^ value for prognosis is medium (>.13). Results are summarized in Table 8 and indicated that aspirational prototypicality was significantly and positively associated with all three outcomes (*β*'s = .48–.75). In contrast, average prototypicality was not associated with positive perceptions or prognosis, and, surprisingly, was negatively associated with expertness (*β* = −.18).

**TABLE 8:** Effect of average and ideal‐type prototypicality on positive perceptions, expertness, prognosis in Study 1.

Prototypicality type	Positive perceptions		Expertness		Prognosis	
	*β* (*SE*)	*p*	*β* (*SE*)	*p*	*β* (*SE*)	*p*
Average	.02 (.03)	.644	−.18 (.06)	.003**	.02 (.03)	.713
Aspirational	.66 (.03)	<.001***	.75 (.04)	<.001***	.48 (.03)	<.001***

*Note*: *N* = 281. Model for expertness bootstrapped 1000 times. Coefficients are standardized betas and can be used to assess the relative importance of predictors. Following general guidelines (Cohen, 1988), we considered *β* = .10 a small effect, *β* = .30 a medium effect and *β* = .50 a large effect.

Abbreviation: *SE*, standard error.

** *p* < .01.

*** *p* < .001.

##### Identification as depressed

We examined whether identification as depressed moderated the effect of condition on positive perceptions, expertness and prognosis using the PROCESS macro for *R* (Model 1; Hayes, 2013). The independent variable was condition (dummy coded so that the control condition was the reference category) and the moderator was identification as depressed. We ran separate analyses for each dependent variable (bootstrapping the expertness model, specifying 1000 repetitions to handle the normality violations). All continuous variables were standardized before analysis. Results are displayed in Table 9. All three models were significant: positive perceptions, *F*(7, 274) = 11.87, *p* < .001, *R* ^2^ = .23; expertness, *F*(7, 274) = 9.94, *p* < .001, *R* ^2^ = .20 and prognosis, *F*(7, 274) = 8.14, *p* < .001, *R* ^2^ = .17. According to Cohen (1988), these *R* ^2^ values all indicate a medium effect size. Identification as depressed had a significant positive main effect on all three outcome variables. The effect of condition varied across outcomes, with a significant negative effect of current depression (vs. control) on expertness, but no significant main effects of condition on positive perceptions or prognosis (as would be expected given the results of H1). There were significant interactions between identification as depressed and condition for positive perceptions and expertness, but not for prognosis. Specifically, for positive perceptions and expertness, participants rated the therapist disclosing current depression more positively to the extent that they identified as depressed.

**TABLE 9:** Effect of identification as depressed on positive perceptions, expertness, and prognosis, by condition in Study 1.

Predictor	Positive perceptions	Expertness	Prognosis
	*β* (*SE*)	*β* (*SE*)	*β* (*SE*)
Identification as depressed (ID)	0.30** (0.11)	0.23* (0.11)	0.46*** (0.11)
Professional experience (vs. Control)	−0.08 (0.15)	−0.04 (0.15)	−0.02 (0.16)
Current depression (vs. Control)	−0.16 (0.16)	−0.41* (0.16)	−0.06 (0.16)
Recovered depression (vs. Control)	0.16 (0.15)	0.01 (0.15)	0.25 (0.16)
ID × Professional experience	0.01 (0.15)	0.04 (0.15)	−0.11 (0.15)
ID × Current depression	0.31* (0.15)	0.39* (0.15)	−0.01 (0.16)
ID × Recovered depression	0.30 (0.16)	0.19 (0.16)	−0.11 (0.16)

*Note*: *N* = 282. Model for expertness bootstrapped 1000 times. Coefficients are standardized betas and can be used to assess the relative importance of predictors. Following general guidelines (Cohen, 1988), we considered *β* = .10 a small effect, *β* = .30 a medium effect, and *β* = .50 a large effect.

Abbreviation: ID, identification as depressed; *SE*, standard error (in parentheses).

* *p* < .05.

** *p* < .01.

*** *p* < .001.

### Study 1 Discussion

Study 1 represented the first attempt to test the effect of therapist lived experience disclosures among real clients and therapists and the first study to consider the effect of such disclosures on therapist prototypicality. The first two hypotheses were not supported: (1) participants in the four conditions did not differ in their ratings of the therapist on any of the outcomes (positive perceptions, expertness and expected prognosis) and (2) therapy clients did not rate disclosing therapists more favourably than therapists. These findings contrast with those observed by Moody et al. (2021) that therapists recounting past mental health conditions were rated more favourably than non‐disclosing therapists or therapists disclosing a current mental health condition. This discrepancy may be at least in part due to differences in the vignette settings (group vs. individual therapy) and samples (real therapists and clients vs. general population) between our study and Moody et al.'s. Our findings also provide some initial evidence that therapists and clients have similar views about therapists disclosing mental health conditions.

Supporting Hypothesis 3, the therapist disclosing recovered depression was rated highest on aspirational prototypicality, and the therapist disclosing current depression was rated highest on average prototypicality. Interestingly though, and contrary to our hypothesis, the therapist disclosing recovered depression was also rated highly on average prototypicality (and similarly to the therapist disclosing current depression). Notably too, exploratory analyses demonstrated that aspirational prototypicality (*β*s = .48–.75) was consistently more strongly associated with positive perceptions of the therapist, ratings of their expertness and expected prognosis than average prototypicality (*β*s = −.18–.02). However, the results of this exploratory analysis should be considered with caution given that the outcomes and the prototypicality types were measured at the same time point. It is thus unclear whether these outcomes led to higher aspirational prototypicality ratings or vice versa, necessitating additional longitudinal research.

There was no evidence that disclosing professional experience led to therapists being perceived as prototypical. In fact, our findings suggest this may even have had a ‘backlash effect’ that led therapists to be rated the lowest on both types of prototypicality, even lower than a non‐disclosing therapist (although not a significant difference). One possible explanation for this is that talking about years of professional experience working with people who have depression may be considered an irrelevant disclosure. Related to this, Knox and Hill (2003) stated that therapists sharing that they have worked with many clients who have experienced an issue being discussed is likely insufficient for building rapport with a client.

Planned exploratory analyses of the effect that participants' connection with depression had on the results revealed that the more participants identified as depressed, the more favourably they rated their positive perceptions, and the expertness, of the therapist disclosing current depression. One explanation for this is that people who identify strongly as depressed are more likely to view a therapist disclosing current depression as an ingroup member. This may lead to more positive views of the therapist, in line with a long history of research suggesting that people tend to view members of their ingroup more favourably than outgroup members (Tajfel, 1982). Interestingly, despite not being part of our eligibility criteria, the majority of both clients and therapists in our sample reported past experience of depression, with many clients also scoring highly on current depression symptoms. Experience with current or past depression did not affect our results. However, the high level of familiarity with depression in our sample may indicate that many participants could relate personally to the depression‐focused vignettes.

## STUDY 2

Study 2 aimed to extend the findings of Study 1 by (a) establishing whether the *relevance* of the lived experience disclosure affected the way it was perceived and (b) comparing the responses of clients and the general population. To this end, Study 2 was identical to Study 1 in most respects, except that we replaced the professional experience condition with disclosure of less relevant recovered lived experience (recovered anxiety) and recruited general population adults rather than therapists for our second subsample. We theorized that the therapist disclosing professional experience in Study 1 may have been rated the least prototypical due to the irrelevance of this disclosure and wanted to determine whether a less relevant recovery disclosure would have a similar effect (i.e., indicating that the disclosure should be recovered and *relevant* to signal prototypicality). Our hypotheses were as follows:

First, we wanted to investigate why Study 1 departed from previous research that found significant differences between how different types of therapist self‐disclosure were received (using the same scale: positive perceptions). We hypothesized that this may be due to the difference in samples: our study recruited clients and therapists, whereas Moody et al. (2021) recruited general population adults. To test this, we recruited samples of both clients and general population adults. We hypothesized a significant interaction between sample (client or general population adult) and condition (the disclosure type that the therapist made) for ratings on the positive perceptions variable, such that only participants in the general population adult sample would rate a therapist disclosing recovered depression more positively than therapists making other disclosures (H4).

Additionally, we sought to provide a more conservative test of the link between recovery disclosures and prototypicality. We hypothesized that a therapist disclosing recovered anxiety would be rated significantly lower on both aspirational and average prototypicality compared to the therapist disclosing recovered depression (H5).

### Study 2 Material and methods

#### Participants

We recruited two subsamples via Prolific: 175 clients who had received therapy within the last year and 177 adults in the general population. For the client subsample, recruitment progressed in the same way as Study 1: 260 respondents completed a paid screener, from which 195 respondents were deemed eligible and invited to complete the full study (175 did). From the standard pool of Prolific participants (excluding any participants who had already participated in the study), we recruited 177 general population adults (two extra participants were recruited due to an error). As in Study 1, all participants completed an attention check and manipulation check. These checks led to the removal of 89 participants (47 clients, 42 general population adults): 79 participants failed the manipulation check (at a similar rate across all conditions), four participants failed the attention check and six participants failed both. Aligned with the standard Prolific rate, participants received £1.25 for their time completing the full survey plus £0.17 for completing the screener.

There were comparable demographic characteristics across the two subsamples (Table 10). All participants resided in the United Kingdom.

**TABLE 10:** Study 2 sample demographics.

Subsample	Mean age (*SD*)	Gender, % (*n*)	Ethnicity, % (*n*)
General population adults (*N* = 135)	37.42 (10.65)	Women = 71.1% (96) Men = 27.4% (37) Rather not say = 1.5% (2)	White = 80.0% (108) Black = 5.9% (8) Asian = 7.4% (10) Mixed = 3.7% (5) Middle Eastern <1% (1) Rather not say = 2.2% (3)
Clients (*N* = 128)	35.44 (9.86)	Women = 65.6% (84) Men = 32.0% (41) Non‐binary = 2.3% (3)	White = 89.1% (114) Black = 5.5% (7) Asian = 3.9% (5) Mixed <1% (1) Middle Eastern <1% (1)

#### Procedure, measures and materials

The procedure and measures used were the same as in Study 1. The only differences were that (a) the professional experience condition was replaced by a condition in which the therapist disclosed recovered anxiety and (b) we did not measure the depression covariates. For the new condition, the last two sentences of the scenario were: *The therapist also tells the group that they have personal experience with anxiety but have now recovered after many years of struggling. The therapist says that this means they really understand how difficult it is to live with depression*.

### Study 2 Results

Of the 263 participants, 17 participants had missing data (6.5%), which was managed using the same approach as Study 1. We used mean comparisons to examine H4 and H5. Assumptions for ANOVA (the primary analysis to test our hypotheses) were tested with Levene's tests, Shapiro–Wilk tests, skewness and kurtosis statistics and inspection of the histograms within each condition and for each dependent variable. These were met with minor exceptions. Levene's tests were non‐significant for all variables (*p*s < .05), and Shapiro–Wilk tests were non‐significant in most cases (*p*s < .05). Where there were significant Shapiro–Wilk tests, this appeared to be due to negative skewness. However, the skewness value was still within the acceptable range of −2 to 2 in all cases (Kim, 2013). As a sensitivity test, all analyses were run as Kruskal–Wallis tests with planned mean comparisons using Dunn's tests. The results were identical, so we report the parametric test results. As in Study 1, we used ANOVA and Chi‐square tests to examine whether there were any baseline differences between participants in the experimental conditions on age, gender and ethnicity. These were non‐significant except for ethnicity, *χ* ^2^(12) = 28.84, *p* = .005. However, we could not add ethnicity as a covariate to our analyses due to several small cell counts (*n* < 2).

Descriptive statistics are summarized in Table 11. We also present descriptive statistics for each measure, split by condition, in Table 12. Consistent with Study 1, none of the mean differences between conditions were significant for positive perceptions, expertness or prognosis, but there were differences for aspirational and average prototypicality (in line with H5; see below).

**TABLE 11:** Descriptive statistics of Study 2 key variables.

Variable	Mean	*SD*	1	2	3	4	5
1. Average prototypicality	4.66	1.28	1				
2. Aspirational prototypicality	5.05	1.29	.59	1			
3. Positive perceptions	3.97	0.63	.43	.67	1		
4. Prognosis for treatment	2.42	0.72	.36	.51	.55	1	
5. Expertness	5.46	1.05	.38	.65	.76	.55	1

*Note*: All correlations are significant, *p* < .001. According to Funder and Ozer (2019), all correlations indicate a large effect size (*r* > .30).

Abbreviation: *SD*, standard deviation.

**TABLE 12:** Means and standard deviations (*SD*s) of Study 2 key outcomes by condition.

Variable	Control		Current depression		Recovered depression		Recovered anxiety	
	Mean	*SD*	Mean	*SD*	Mean	*SD*	Mean	*SD*
Positive perceptions	3.98	0.59	3.89	0.66	4.06	0.63	3.93	0.65
Expertness	5.55	1.02	5.36	1.05	5.61	0.99	5.27	1.14
Prognosis	2.38	0.72	2.34	0.70	2.57	0.76	2.36	0.68
Aspirational prototypicality	4.60^a^	1.24	4.88^a^	1.5	5.60^b^	1.12	4.99^a^	1.21
Average prototypicality	3.67^c^	1.13	5.38^d^	0.99	5.01^d^	1.10	4.41^e^	1.21

*Note*: Means with different superscript letters differ significantly, *p* < .05.

#### H4: Client versus general population ratings

We conducted a two‐way ANOVA with positive perceptions as the outcome and condition, sample and their interaction as predictors. The focal test for the hypothesis was the significance of the interaction coefficient, indicating differences between subsamples in the effect of therapist disclosure condition on perceptions. Results did not support the hypothesis; the interaction was nonsignificant (as were the main effects; see Table 13). Additional analyses indicated that there were also no significant interactions for expertness or prognosis. This suggests that participants in both samples viewed the different disclosures similarly.

**TABLE 13:** Effect of condition (disclosure type) and sample on positive perceptions in Study 2.

Model term	*df*	Condition		
		*F*	*p*	*η* ^2^
Condition	3, 255	0.986	.400	.01
Sample	1, 255	2.316	.129	.01
Condition × Sample	3, 255	0.927	.428	.01

*Note*: *η* ^2^ reported are partial. According to Richardson (2011), these all indicate a small effect.

#### H5: Effect of recovered anxiety disclosure

Two one‐way ANOVAs with prototypicality (aspirational or average) as the outcome and condition as the predictor were used to test H5. Both ANOVAs were significant (see Table 14 for results and Table 12 for mean comparisons). Supporting H5, the therapist disclosing recovered anxiety was rated significantly lower on both aspirational and average prototypicality compared to the therapist disclosing recovered depression.

**TABLE 14:** Effect of condition (disclosure type) on prototypicality in Study 2.

Prototypicality type	*df*	Condition		
		*F*	*p*	*η* ^2^
Aspirational	3, 257	8.527	<.001***	.09
Average	3, 259	29.968	<.001***	.26

*Note*: *N* = 260 for aspirational model; *N* = 262 for average model. According to Cohen (1988), the effect size for *η* ^2^ can be interpreted such that *η* ^2^ = .01 is a small effect, *η* ^2^ = .06 is a medium effect, and *η* ^2^ = .14 is a large effect.

*** *p* < .001.

### Study 2 Discussion

Study 2 extended the findings of Study 1. Interestingly, and not supporting Hypothesis 4, ratings of the different disclosures on the outcomes were similar between the client and general population samples. This provides preliminary evidence that clients view therapist self‐disclosure of lived experience similarly to general population adults, suggesting that prior research recruiting general population samples may accurately capture the perspectives of clients. This also suggests our findings that depart from Moody et al. (2021) may simply be due to the use of a group therapy scenario rather than an individual therapy scenario, or the use of a United Kingdom versus United States sample, highlighting the possible importance of the broader context on how therapist self‐disclosures are perceived. We also examined whether less relevant recovery disclosures would still allow the therapist to be viewed as prototypical, or whether it had to be the same mental health condition experienced by the therapy group. Supporting Hypothesis 5, results indicated that disclosing a recovered and *relevant* condition was key to being viewed as aspirationally prototypical. By contrast, *any* lived experience disclosure (whether recovered or current, irrelevant or relevant) led to therapists being viewed as prototypical of an average group member (compared to no disclosure), with maximally positive effects when the disclosure was of the same condition experience by the clients (recovered or current depression).

## GENERAL DISCUSSION

We aimed to examine how therapists were perceived by clients, other therapists and adults in the general population depending on what kind of lived experience disclosure they made. We tested this in the context of group therapy for depression and were specifically interested in whether therapists were rated more positively if they disclosed that they had recovered from depression, compared to disclosing: (a) current depression, (b) recovered anxiety, (c) professional experience with depression or (d) nothing. Contrary to several of our preregistered hypotheses, we found no differences across conditions or samples in how participants rated the therapists on our key outcomes of interest (positive perceptions, expertness, prognosis). Specifically, in Study 1, we found no support for our first hypothesis that different types of disclosure would affect positive perceptions of the therapist, ratings of their expertness or expected prognosis, nor for our second hypothesis that clients and therapists would rate disclosures differently. Similarly, in Study 2, our fourth hypothesis predicting differences in how clients and general population adults would view the disclosures was not supported and we again found no difference across the positive perceptions, expertness and prognosis outcomes between conditions. This provides evidence that neither experience of therapy, nor working as a therapist, necessarily leads people to hold different perspectives on therapist self‐disclosure and raises interesting questions about how views on therapist self‐disclosure might be shaped by broader societal understandings of therapeutic relationships rather than specific therapeutic experiences.

The limited support for H1 was also unexpected given that, in a study using a similar design, Moody et al. (2021) found that therapists disclosing a condition from the distant past were rated more favourably than therapists disclosing a current condition. As mentioned in the Study 1 Discussion, these differences may reflect our use of a group rather than an individual therapy vignette (which Moody et al., 2021 used). Another possible explanation for these contrasting findings is that there may be cultural differences in attitudes towards therapist self‐disclosure between the United States (where many of the participants in Moody et al.'s study likely resided given their use of the MTurk recruitment platform) and the United Kingdom (where most of our participants resided).

We found more support for our hypotheses derived from identity leadership theory. In Study 1, H3a was supported: participants rated therapists who disclosed recovered depression higher on aspirational prototypicality compared to other therapists. Extending this, in Study 2, H5 was also supported, such that the therapist disclosing recovered depression was rated higher on aspirational and average prototypicality than the therapist disclosing recovered anxiety. This indicates that, to signal aspirational prototypicality, disclosures should be of recovered *and relevant* conditions. These findings help to provide a new theoretical reason for the previous finding that the similarity between what therapists disclose and the client's experience has important consequences for therapeutic outcomes (Levitt et al., 2016). That is, relevant disclosures may be more impactful because they are uniquely able to signal a therapist's aspirational prototypicality.

Interestingly, across both studies, recovered depression disclosures were rated similarly to current depression disclosures on average prototypicality (‘who we are’). This was contrary to H3b. Disclosure of recovered depression, therefore, allowed therapists to represent both ‘who we are now’ and ‘who we want to be’ for clients. Comparatively, other types of lived experience disclosure only allowed therapists to represent ‘who we are now’. Although we had hypothesized otherwise, our findings suggest that recovering from a condition may not necessarily diminish a therapist's perceived ability to understand clients' current experiences of that condition. This finding has important implications for identity leadership theory, as previous studies in this area have tended to examine whether leaders embody average *or* aspirational prototypicality (e.g., Robertson et al., 2022; Steffens et al., 2021), rather than considering the possibility that leaders may embody both types simultaneously. Future research should continue to consider the possibility that behaviours which signal aspirational prototypicality (such as recovery disclosures) might also involve signalling some degree of average prototypicality.

By demonstrating the benefits of recovery disclosures for signalling aspirational prototypicality (which has been linked to positive therapeutic outcomes; Robertson et al., 2022), our findings also provide support for the growing ‘lived experience movement’ in mental health care research and delivery (Sunkel & Sartor, 2022) and suggest that therapists should not be overlooked as a source of lived experience (as they often are, despite the high prevalence of current or past mental health conditions in this population; de Vos et al., 2016; Victor et al., 2022). However, we demonstrate that the positive effects of lived experience disclosures may be maximized when therapists discuss recovered, rather than current, lived experience. This speaks to the appropriateness of the recommendation that therapists should not disclose experiences that are still currently negatively affecting them (Gelso & Palma, 2011; Henretty & Levitt, 2010; Knox & Hill, 2003).

### Strengths, limitations and future research

Our studies had several strengths, including a strong theoretical underpinning, novelty (investigation of self‐disclosure in a group, rather than individual, therapy context), use of an experimental design and recruitment of diverse samples. Nevertheless, our findings should be considered in light of some limitations. Perhaps most saliently, we examined therapist self‐disclosure using vignettes, rather than in a natural therapy setting. Research in real‐world therapy contexts is required to evaluate the ecological validity of these findings. Such research has some key barriers. For example, there would be ethical concerns associated with using experimental designs in which some therapists are asked to disclose mental health conditions during therapy while others are not. Although it would lack the ability to make causal inferences, future research could compare the extent to which clients perceive their therapists engage in disclosure during real therapy sessions and assess the relationships between this and key therapy outcomes.

The cross‐sectional nature of our study also limited our ability to examine whether prototypicality mediated the relationship between therapist disclosure types and our outcome variables (perceptions of the therapist, ratings of expertness and expected prognosis). When considered alongside our finding that disclosure type affects prototypicality ratings, our exploratory findings regarding the association between prototypicality and outcomes speak to a potential mediation effect. However, longitudinal research assessing both the prototypicality types and the outcomes at multiple time points would be required to provide insight into the direction of the relationships between these variables (noting evidence from other contexts that relationships between identity leadership and positive outcomes may be bidirectional; Bruner et al., 2022).

Additionally, there are limitations associated with our sampling approach. As mentioned, we did not specifically recruit participants with depression. Even though our Study 1 sample demonstrated relatively high depression symptoms and identification with depression, suggesting they could likely relate to the therapy scenarios presented, this would still have varied across participants. Future research specifically targeting participants with depression would therefore be beneficial, particularly given identity leadership theory's emphasis on the importance of connecting over shared identities.

Finally, we recruited participants who had received or delivered broadly defined ‘mental health services’ in the previous year and did not collect information about participants' therapeutic experiences. Moreover, although we collected information about the role that the therapists worked in, we did not ask client participants about the clinician who provided mental health care to them. These broad inclusion criteria may have resulted in a heterogeneous sample with varying levels and types of therapeutic engagement, which may have affected how participants viewed the scenarios. For example, some clients may have been deemed eligible due to seeing peer workers—individuals who are known to commonly share their lived experience with clients (Jacobson et al., 2012). Where practical, future research should aim to minimize differences between the type of mental health care accessed by clients and delivered by therapists. This research could also seek to compare attitudes across, for example, different health professionals (e.g., psychologists vs. general practitioners).

## CONCLUSIONS

Across two experiments, we examined how different types of therapist lived experience disclosures were perceived in the context of group therapy for depression. Contrary to our expectations and some previous research, we found no evidence that different types of disclosure directly affected participants' positive perceptions of the therapist, ratings of the therapist's expertness or expected prognosis for treatment. Additionally, there were no differences in how clients, therapists or general population adults rated the different disclosures on these outcomes. We obtained more support for our hypotheses drawn from identity leadership theory. Our results suggested that the content of disclosure matters for determining whether a therapist is viewed as a prototypical member of the therapy group. Specifically, therapists who disclosed a *recovered and relevant* condition were seen to represent both ‘who we are now’ (average prototypicality) and ‘who we want to be’ (aspirational prototypicality). In contrast, disclosure of less relevant or current conditions signalled average, but not aspirational, prototypicality. This is important to consider in light of evidence that group therapy facilitators are more effective to the extent they are seen as aspirationally (rather than averagely) prototypical. Our findings extend on this prior research by highlighting one way that therapists can signal their aspirational prototypicality: by disclosing information about their own relevant prior experiences.

## AUTHOR CONTRIBUTIONS

**Alysia M. Robertson:** Conceptualization; methodology; formal analysis; investigation; writing – original draft; writing – review and editing; visualization. **Tegan Cruwys:** Conceptualization; methodology; supervision; writing – original draft; writing – review and editing. **Mark Stevens:** Conceptualization; methodology; supervision; writing – original draft; writing – review and editing. **Michael J. Platow:** Conceptualization; methodology; supervision; writing – original draft; writing – review and editing.

## CONFLICT OF INTEREST STATEMENT

None.

## Data Availability

The data that support the findings of this study are openly available in OSF at https://osf.io/az2v9/.
